# Combined prognostic value of pretreatment anemia and cervical node necrosis in patients with nasopharyngeal carcinoma receiving intensity‐modulated radiotherapy: A large‐scale retrospective study

**DOI:** 10.1002/cam4.1233

**Published:** 2017-10-16

**Authors:** Lu‐Lu Zhang, Guan‐Qun Zhou, Yi‐Yang Li, Ling‐Long Tang, Yan‐Ping Mao, Ai‐Hua Lin, Jun Ma, Zhen‐Yu Qi, Ying Sun

**Affiliations:** ^1^ Department of Radiation Oncology Sun Yat‐sen University Cancer Center State Key Laboratory of Oncology in South China Collaborative Innovation Center for Cancer Medicine 651 Dongfeng Road East Guangzhou 510060 China; ^2^ Department of Oncology the First affiliated Hospital of Guangdong Pharmaceutical University Guangdong 510080 China; ^3^ Department of Medical Statistics and Epidemiology School of Public Health Sun Yat‐sen University Guangzhou China

**Keywords:** Anemia, cervical node necrosis, nasopharyngeal carcinoma, survival

## Abstract

This study investigated the combined prognostic value of pretreatment anemia and cervical node necrosis (CNN) in patients with nasopharyngeal carcinoma (NPC). Retrospective review of 1302 patients with newly diagnosed nonmetastatic NPC treated with intensity‐modulated radiotherapy (IMRT) ± chemotherapy. Patients were classified into four groups according to anemia and CNN status. Survival was compared using the log‐rank test. Independent prognostic factors were identified using the Cox proportional hazards model. The primary end‐point was overall survival (OS); secondary end‐points were disease‐free survival (DFS), locoregional relapse‐free survival (LRRFS), and distant metastasis‐free survival (DMFS). Pretreatment anemia was an independent, adverse prognostic factor for DMFS; pretreatment CNN was an independent adverse prognostic factor for all end‐points. Five‐year survival for non‐anemia and non‐CNN, anemia, CNN, and anemia and CNN groups were: OS (93.1%, 87.2%, 82.9%, 76.3%, *P *<* *0.001), DFS (87.0%, 84.0%, 73.9%, 64.6%, *P *<* *0.001), DMFS (94.1%, 92.1%, 82.4%, 72.5%, *P *<* *0.001), and LRRFS (92.8%, 92.4%, 88.7%, 84.0%, *P *=* *0.012). The non‐anemia and non‐CNN group had best survival outcomes; anemia and CNN group, the poorest. Multivariate analysis demonstrated combined anemia and CNN was an independent prognostic factor for OS, DFS, DMFS, and LRRFS (*P *<* *0.05). The combination of anemia and CNN is an independent adverse prognostic factor in patients with NPC treated using IMRT ± chemotherapy. Assessment of pretreatment anemia and CNN improved risk stratification, especially for patients with anemia and CNN who have poorest prognosis. This study may aid the design of individualized treatment plans to improve treatment outcomes.

## Introduction

Nasopharyngeal carcinoma (NPC) is a malignancy of the nasopharyngeal epithelium. Although NPC is rare globally, it is highly prevalent in southern China where the incidence is 20 to 50 cases per 100,000 males 1. Radiotherapy (RT) is the primary therapy for nonmetastatic NPC 2, 3. The tumor‐node‐metastasis (TNM) staging system for NPC is the most powerful tool for guiding the selection of treatment strategies and predicting the prognosis of patients with NPC 4. Although application of superior RT technique intensity‐modulated radiation therapy (IMRT) and addition of chemotherapy and the precise imaging technology magnetic resonance imaging (MRI) have improved locoregional control, the survival outcomes among patients with advanced NPC remain unsatisfactory 5, 6, 7. Therefore, the identification of clinically relevant prognostic factors to recognize patients at high‐risk of failure is necessary.

Radioresistance and chemoresistance are the main factors leading to distant metastasis and tumor progression 8. Tumor hypoxia is key factor in development of radio‐ and chemo‐resistance 9, 10. Numerous efforts have been made to identify tumor hypoxia‐related prognostic factors for NPC in recent years 11, 12, 13, 14, 15, 16, 17, 18, 19, 20; hemoglobin (Hb) levels and cervical node necrosis (CNN) have attracted significant attention. Hb is the principal carrier of oxygen in red blood cells, which carry oxygen from the respiratory organs to the rest of the body. A low Hb level causes a reduction in blood oxygen and can lead to tumor hypoxia. CNN may indicate intratumoral hypoxia 21. Anemia or/and CNN may in turn affect the efficacy of RT and chemotherapy. Accumulating data suggests there are positive relationships between pretreatment hemoglobin (Hb) levels and CNN and the survival of patients with NPC treated with IMRT ± chemotherapy 11, 12, 13, 14, 15, 16, 17. As both anemia and CNN may serve as markers of low tumor oxygenation status, it is reasonable to hypothesize the pretreatment hemoglobin level may correlate with and complement CNN to enable improved prediction of the survival of patients with NPC. However, this relationship has not been confirmed experimentally.

We performed this retrospective study to evaluate the impacts of pretreatment anemia and CNN on survival outcomes and then investigated the combined prognostic value of these factors, with the aim of improving outcome prediction and enabling the elucidation of individualized strategies for treatment of patients with NPC undergoing IMRT.

## Materials and Methods

### Patient selection and staging evaluation

The inclusion criteria were: (1) had newly diagnosed, pathologically proven, previously untreated NPC; (2) with cervical node metastasis; (3) no evidence of distant metastases; (4) treated using IMRT ± chemotherapy at our cancer center from November 2009 to February 2012; (5) no other tumors or serious illnesses; (6) available pretreatment MRI scans of the nasopharynx and neck; (7) pretreatment Hb level available (measured ≤1 week before treatment); (8) and no other tumors or serious illnesses. The selection criteria were met by 1302 consecutive patients. Participants completed pretreatment baseline evaluations including a complete history, hematological and biochemical profiles, physical and neurological examination, chest radiography, MRI of the neck and nasopharynx, abdominal ultrasonography, and a whole‐body bone scan using single photon emission computed tomography. All participants were restaged based on the seventh edition of the International Union against Cancer/American Joint Committee on Cancer system 22. Patient records/information were collected by trained data reviewers and were analyzed anonymously. As this study was a retrospective assessment of routine data, the ethics committee of our Cancer Center waived the need for individual informed consent.

### Treatment strategies

Treatment strategies were designed using standard protocols depending on clinical TNM stage and the general patient condition. In the relevant study period, institutional guidelines recommended concurrent chemoradiotherapy ± neoadjuvant/adjuvant chemotherapy for stage II to IVB as described previously 23. Every patient was treated with IMRT at one fraction/day, 5 days per week. IMRT was performed as described previously 23.

Concurrent chemotherapy consisted of cisplatin weekly or every 3 weeks during RT. Neoadjuvant/adjuvant chemotherapy was 1–4 cycles of cisplatin with docetaxel, cisplatin with 5‐fluorouracil or cisplatin with 5‐fluorouracil and docetaxel every 2 to 3 weeks.

### Assessment of anemia and CNN

Anemia was defined based on the Chinese Society of Clinical Oncology (CSCO) clinical practice guidelines and National Cancer Institute criteria on cancer‐related anemia: pretreatment hemoglobin <120 g/L in males and <110 g/L in females 21, 24, 25.

All patients underwent MRI using a 1.5‐T MR system (Signa CV/i; General Electric Healthcare, Chalfont St. Giles, United Kingdom) using a head and neck combined coil to examine the region from the suprasellar cistern to the inferior margin at the sternal end of the clavicle. Two radiologists specializing in HN cancer reviewed all MRI scans separately and any disagreements were resolved by discussion every 2 weeks. The definition of necrosis on MRI was T1‐weighted contrast‐enhanced images showing focal low‐signal intensity with or without a surrounding rim of enhancement or T2‐weighted images showing focal high‐signal intensity.

Patients were classified into four groups: non‐anemia and non‐CNN group, anemia group (anemia and non‐CNN group), CNN group (non‐anemia and CNN group), and anemia and CNN group.

### Follow‐up and end‐points

Follow‐up time was measured from day 1 of treatment to the time of last visit or death. To detect possible locoregional relapse or distant metastasis, all patients underwent conventional examinations (similar to the pretreatment assessment) every 3 to 6 months during the first 24 months, and 6‐monthly thereafter until death. End‐points (from first day of treatment to first defining event) were overall survival (OS), disease‐free survival (DFS), distant metastasis‐free survival (DMFS), and locoregional relapse‐free survival (LRRFS).

### Statistical analysis

Data analysis used Statistical Package for the Social Sciences version 17.0 software (SPSS, Chicago, IL, USA); Survival rates were calculated using the Kaplan–Meier method, and survival curves were compared using log‐rank tests. Clinical characteristics and failure patterns between groups were compared using the Chi‐square test or Fisher's exact test. If more than 20% of the expected count in cells is less than 5, use the Fisher's exact probability tests, otherwise use the Pearson chi‐square test. Univariate analysis was conducted using the log‐rank test. Multivariate analysis used the Cox proportional hazards model including the following covariates: gender, age, T category, N category, WHO histological type, presence or absence of pretreatment anemia, presence or absence of CNN, and chemotherapy (yes/no). Two‐tailed *P*‐values < 0.05 were considered significant.

## Results

### Patient characteristics

At diagnosis, the median age was 45 years (range, 14–78 years). A total of 178 patients (13.7%) were lost to follow‐up by the final follow‐up. Of the 1302 patients, 330 (25.3%) were female and 972 (74.7%) were male; 68 (5.2%) patients had type I or II disease and 1234 (94.8%) had type III disease; 291 (22.4%), 681 (52.3%), and 330 (25.3%) had stage II, III, IV NPC, respectively; and 1193/1302 (91.6%) patients received chemotherapy.

Mean pretreatment Hb value was 142 g/L (range, 82–177 g/L) and 6.8% (89/1302) and 34.4% (448/1302) of patients had pretreatment anemia and CNN, respectively. Of the 1302 patients, 801 (61.5%) did not have pretreatment anemia or CNN; 53 (4.1%) had pretreatment anemia but not CNN; 412 (31.6%) had CNN but not pretreatment anemia; and 36 (2.8%) had pretreatment anemia and CNN.

There was no significant difference in the distribution of age (*P *=* *0.459), histological type (*P *=* *0.627), chemotherapy strategies (*P *=* *0.461) or T category (*P *=* *0.197) when the patients were stratified by anemia; however, significant differences were observed in terms of sex (*P *=* *0.004) and N category (*P *=* *0.026). There was no significant difference in terms of age (*P *=* *0.826), sex (*P *=* *0.498) and histological type (*P *=* *0.867) when the patients were stratified by CNN. However, significant differences were observed in terms of T category (*P *=* *0.048), N category (*P *<* *0.001), and chemotherapy strategies (*P *=* *0.001). The pretreatment clinicopathological characteristics of the 1302 patients are listed in Table 1.

**Table 1:** Patient characteristics and treatment factors (*n *=* *1302)

	Non‐anemia	Anemia	*P*	Non‐CNN	CNN	*P*
Characteristic						
Age (years)			0.407			0.512
≤45	614	41		424	231	
˃45	599	48		430	217	
Gender			0.001			0.257
Male	919	53		646	326	
Female	294	36		208	122	
WHO pathology classification			0.492			0.968
Type I	7	1		5	3	
Type II	54	6		40	20	
Type III	1152	82		809	425	
T category^a^			0.062			<0.001
T1	200	10		152	58	
T2	193	7		130	70	
T3	625	53		464	214	
T4	195	19		108	106	
N category^a^			0.019			<0.001
N1	851	49		632	268	
N2	237	24		148	113	
N3	125	16		74	67	
Overall stage^a^			0.001			<0.001
II	282	9		212	79	
III	636	45		472	209	
IV	295	35		170	160	
Chemotherapy			0.331			0.001
No	104	5		88	21	
Yes	1109	84		766	427	

CNN, cervical node necrosis; WHO, World Health Organization.

a According to the seventh edition of the American Joint Committee on Cancer.

### Treatment outcomes for the entire cohort

The median follow‐up duration was 47.8 months (range, 1.3–75.3 months) for the entire cohort and 50.40 (range, 1.3–75.3 months) for the surviving patients. For the entire cohort, the 5‐year OS, DFS, DMFS, and LRRFS rates were 86.0%, 77.5%, 86.8%, 93.1%, and 90.0%, respectively. During follow‐up, 185/1302 patients (14.2%) developed tumor progression: 115 (8.8%) and 159 (12.2%) developed local regional recurrence and distant metastases, respectively. By last follow‐up, 153 patients had died; the majority (87.5%) of these deaths were attributed to NPC, and 19 (12.5%) had died of other causes.

### Prognostic value of pretreatment anemia and CNN

**Figure 1:**
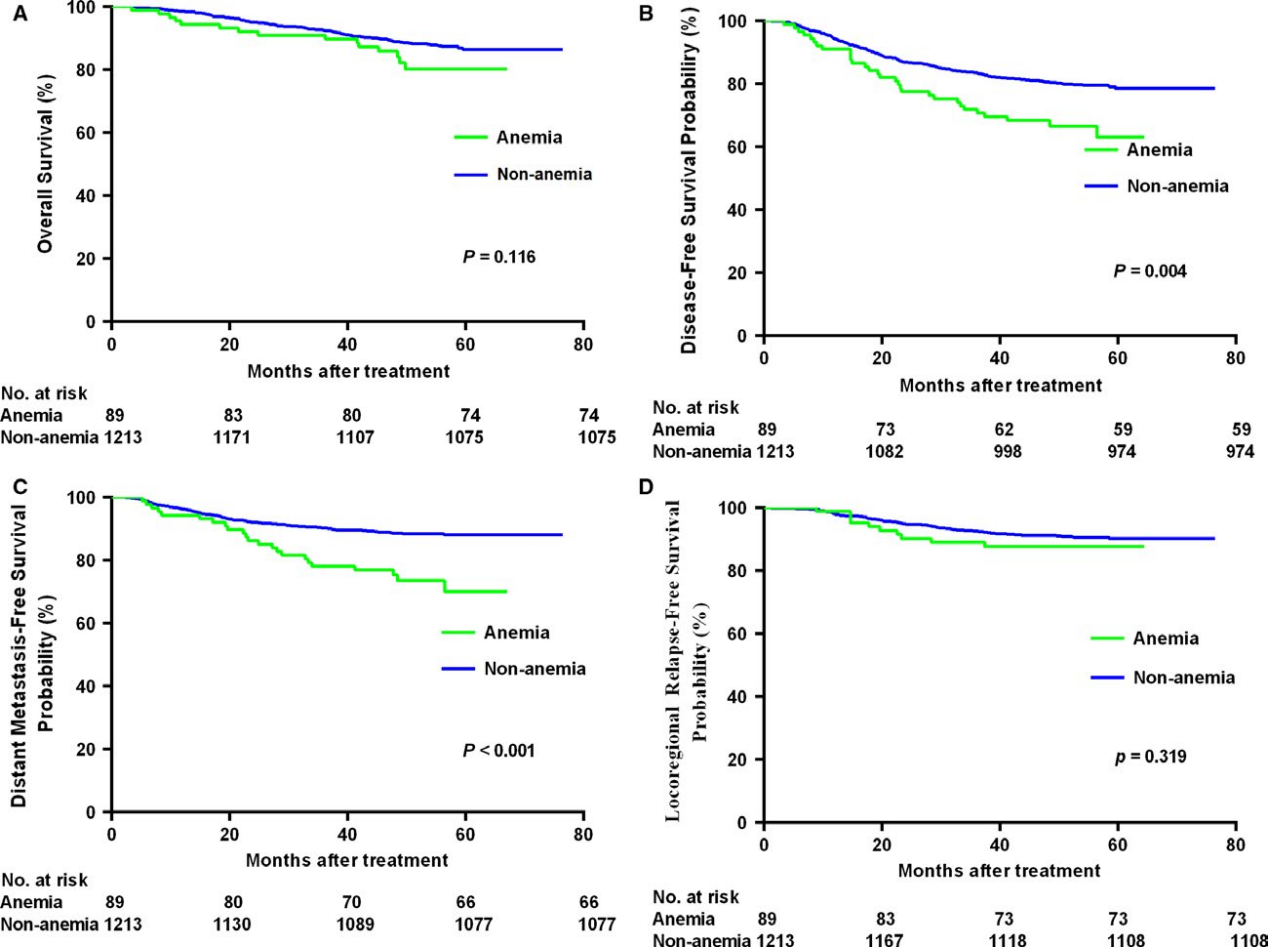
Kaplan–Meier curves of overall (A), disease‐free (B), distant metastasis‐free (C), and locoregional relapse‐free (D) survival outcomes for the 1302 patients with NPC stratified by anemia.

Patients without pretreatment anemia had significantly better 5‐year DMFS and DFS than patients with pretreatment anemia (88.0% vs.70.1%, *P *<* *0.001; 78.6% vs. 63.1%, *P *=* *0.002), though 5‐year OS and 5‐year LRRFS were not significantly different between these groups (86.4% vs. 80.3%, *P *=* *0.116; 90.2% vs. 87.7%, *P *=* *0.319; Fig. 1). In multivariate analysis, after adjusting for various prognostic factors, pretreatment anemia was demonstrated to be an independent prognostic factor for DMFS (HR = 1.73, 95% CI: 1.01–2.70; *P *=* *0.048), but not OS, DFS or LRRFS. In addition, T category (HR = 1.96, 95% CI: 1.06–3.64; *P *=* *0.033) and N category (HR = 2.23, 95% CI: = 1.57–3.17; *P *<* *0.001) were independent prognostic factor for DMFS. More information about the multivariate analyses is given in Table 2 **.**

**Table 2:** Prognostic value of anemia for OS, DFS, DMFS, and LRRFS in the 1302 patients with NPC

Endpoint	Variable	HR (95% CI)	*P*‐value^a^
OS	Anemia	NS	
	WHO pathology	0.33 (0.20–0.55)	<0.001
	T category	3.78 (1.66–8.59)	0.002
	N category	1.80 (1.25–2.60)	0.002
	Age	1.97 (1.36–2.85)	<0.001
	Gender	NS	
	Chemotherapy	NS	
DFS	Anemia	NS	
	WHO pathology	0.47 (0.30–0.75)	0.001
	T category	2.16 (1.35–3.50)	0.001
	N category	1.55 (1.72–2.05)	0.002
	Age	1.46 (1.12–1.90)	0.005
	Gender	NS	
	Chemotherapy	NS	
DMFS	Anemia	1.73 (1.01–2.70)	0.048
	WHO pathology	NS	
	T category	1.96 (1.06–3.64)	0.033
	N category	2.23 (1.57–3.17)	<0.001
	Age	NS	
	Gender	NS	
	Chemotherapy	NS	
LRRFS	Anemia	NS	
	WHO pathology	0.33 (0.18–0.59)	<0.001
	T category	2.25 (1.14–4.46)	0.020
	N category	NS	
	Age	NS	
	Gender	NS	
	Chemotherapy	NS	

NPC, nasopharyngeal carcinoma; HR, hazard ratio; CI, confidence interval; OS, overall survival; LRRFS, locoregional relapse‐free survival; DMFS, distant metastasis‐free survival; DFS, disease free survival; NS, not significant.

a The following parameters were included in the Cox proportional hazards model multivariate analysis: WHO pathology (Type I‐II vs. Type III), gender (male vs. female), age (>45 vs. ≤45 years), chemotherapy (yes vs. no), T category (T1–2 vs. T3–4), and N category (N1 vs. N2–3).

**Figure 2:**
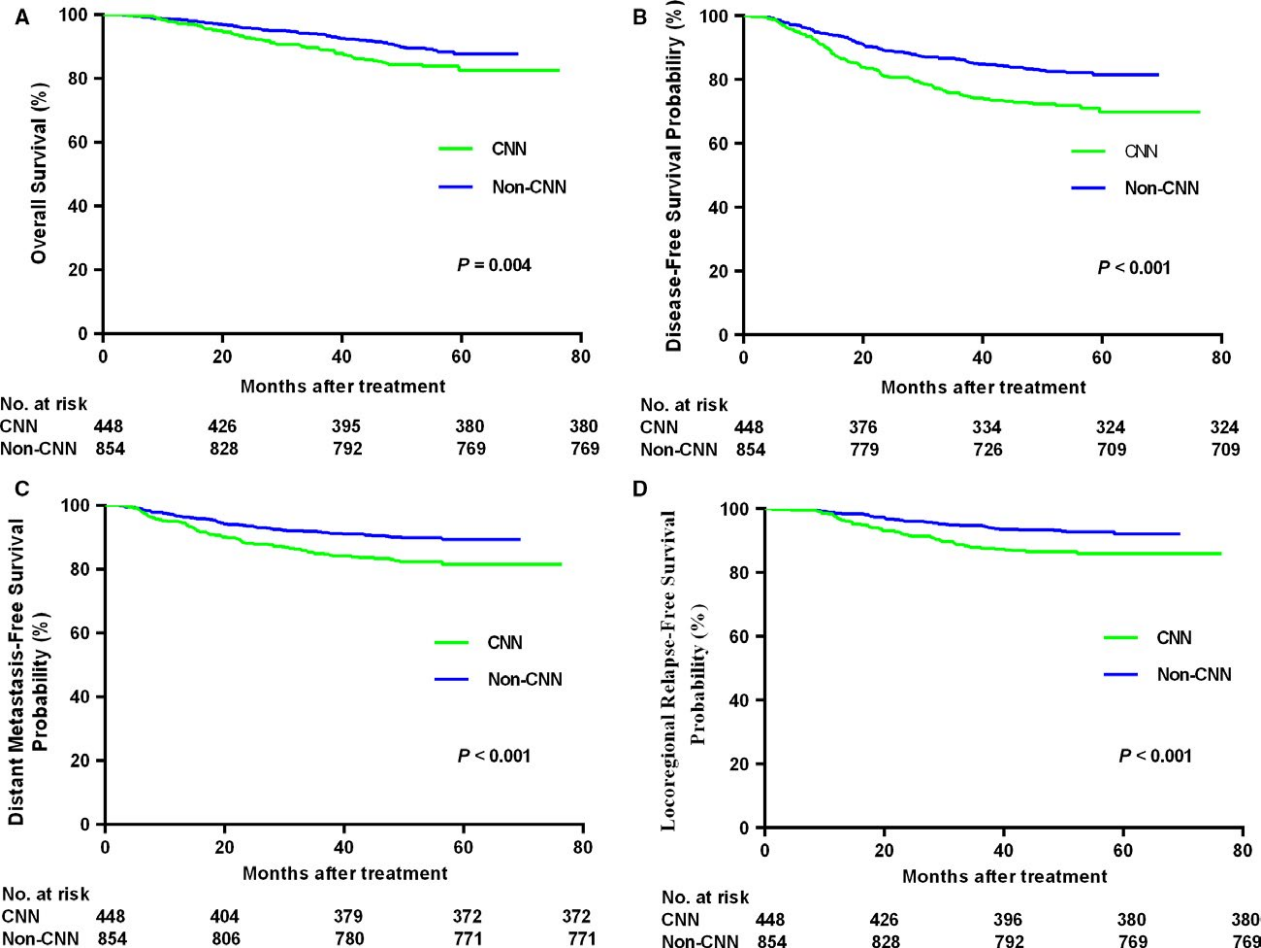
Kaplan–Meier curves of overall (A), disease‐free (B), distant metastasis‐free (C), and locoregional relapse‐free (D) survival outcomes for the 1302 patients with NPC stratified by cervical node necrosis (CNN).

Patients with CNN had significantly poorer 5‐year OS (82.6% vs. 87.8%, *P *=* *0.004), DFS (69.9% vs. 81.6%, *P *<* *0.001), LRRFS (86% vs. 92.1%, *P *<* *0.001) and DMFS (81.6% vs. 89.5%, *P *<* *0.001) than patients without CNN (Fig. 2). Multivariate analyses demonstrated CNN was an independent prognostic factor for OS (HR = 1.50, 95% CI: 1.04–2.15; *P *=* *0.030), DFS (HR = 1.81, 95% CI: 1.31–3.36; *P *<* *0.001), DMFS (HR = 1.70, 95% CI: 1.20–2.41; *P *=* *0.003) and LRRFS (HR = 2.20, 95% CI: 1.50–3.21; *P *<* *0.001). In addition, WHO pathology, T category, N category and age were significant prognostic factor for OS and DFS; T category and N category were independent prognostic factors for DMFS; WHO pathogory and T category were independent prognostic factors for LRRFS. More information about the multivariate analyses is given in Table 3.

**Table 3:** Prognostic value of cervical node necrosis (CNN) for OS, DFS, DMFS and LRRFS in the 1302 patients with NPC

Endpoint	Variable	HR (95% CI)	*P‐*value^a^
OS	WHO pathology	0.33 (0.20–0.59)	<0.001
	CNN	1.50 (1.04–2.15)	0.030
	T category	3.69 (1.62–8.38)	0.002
	N category	1.67 (1.14–2.43)	0.008
	Age	1.47 (1.13–1.91)	<0.001
	Gender	NS	
	Chemotherapy	NS	
DFS	WHO pathology	0.48 (0.31–0.76)	0.002
	CNN	1.81 (1.31–3.36)	<0.001
	T category	2.10 (1.31–3.36)	0.002
	N category	1.42 (1.07–1.89)	0.015
	Age	1.47 (1.13–1.91)	0.004
	Gender	NS	
	Chemotherapy	NS	
DMFS	WHO pathology	NS	
	CNN	1.70 (1.20–2.41)	0.003
	T category	1.92 (1.03–3.57)	0.039
	N category	2.11 (1.48–3.00)	<0.001
	Age	NS	
	Gender	NS	
	Chemotherapy	NS	
LRRFS	WHO pathology	0.33 (0.18–0.58)	<0.001
	CNN	2.20 (1.50–3.21)	<0.001
	T category	2.09 (1.06–4.14)	0.035
	N category	NS	
	Age	NS	
	Gender	NS	
	Chemotherapy	NS	

NPC, nasopharyngeal carcinoma; HR, hazard ratio; CI, confidence interval; OS, overall survival; LRRFS, locoregional relapse‐free survival; DMFS, distant metastasis‐free survival; DFS, disease free survival; NS, not significant.

a The following parameters were included in the Cox proportional hazards model multivariate analysis: WHO pathology (Type I‐II vs. Type III), gender (male vs. female), age (>45 vs. ≤45 years), chemotherapy (yes vs. no), T category (T1–2 vs. T3–4), and N category (N1 vs. N2–3).

### Combined prognostic value of pretreatment anemia and CNN

**Figure 3:**
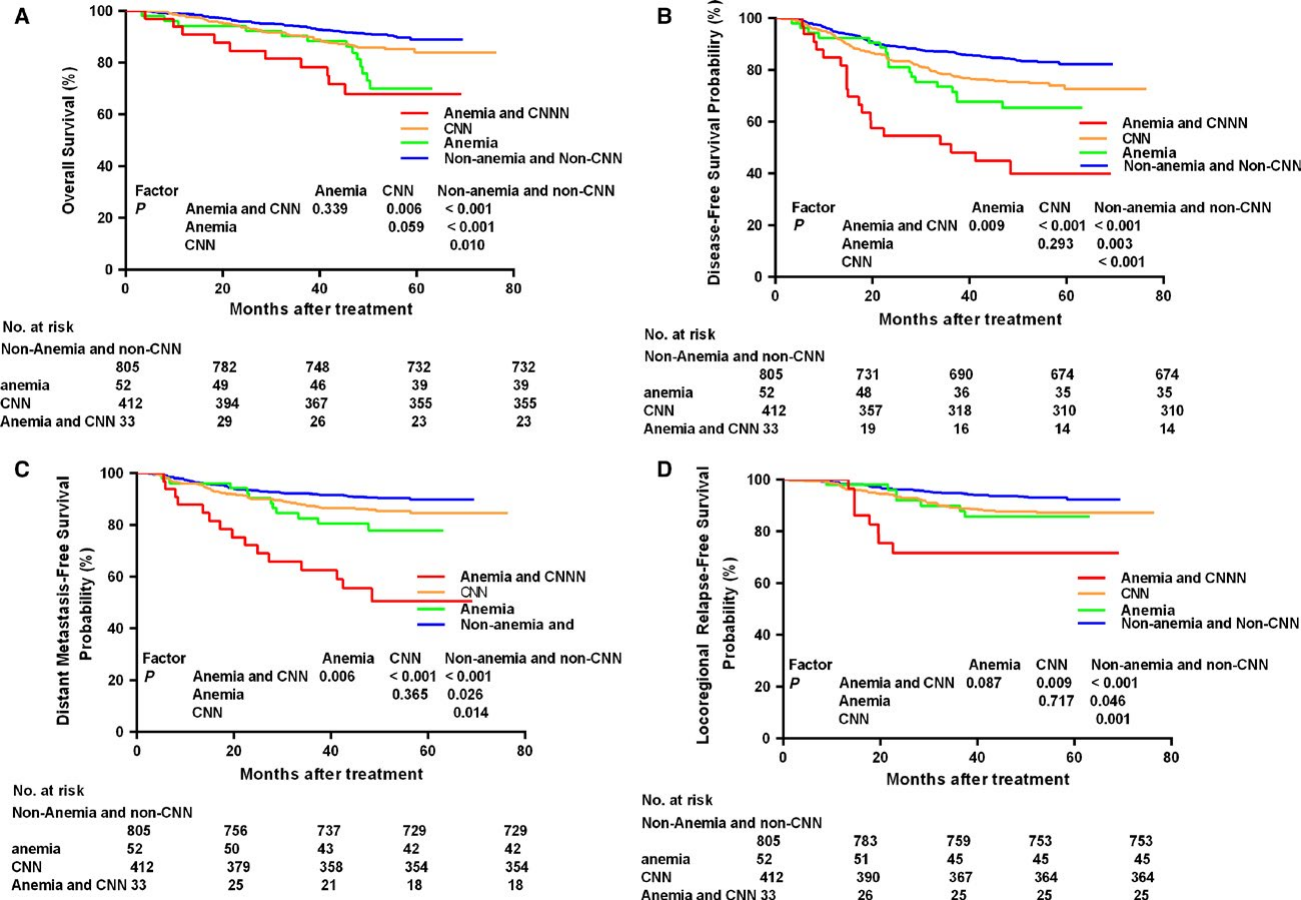
Kaplan–Meier curves of overall (A), disease‐free (B), distant metastasis‐free (C), and locoregional relapse‐free (D) survival outcomes for the 1302 patients with NPC stratified by anemia combined with cervical node necrosis (CNN).

We classified the entire cohort into four groups using the two prognostic factors pretreatment anemia and CNN, as described above. Significant differences in OS, PFS, LRRFS, and DMFS were observed between the non‐anemia and non‐CNN group, anemia group, CNN group, and anemia and CNN groups: 5‐year OS was 88.9%, 70.1%, 84.0%, and 67.9% (*P *<* *0.001); 5‐year DFS was 82.2%, 66.6%, 72.8%, and 39.9% (*P *<* *0.001); 5‐year DMFS was 89.8%, 79.5%, 84.6%, and 50.6% (*P *<* *0.001), and 5‐year LRRFS was 92.3%, 85.5%, 87.2%, and 71.8% (*P *<* *0.001), respectively. Kaplan–Meier survival curves for the four groups are shown in Figure 3.

The anemia and CNN group had the poorest survival outcomes, and the non‐anemia and non‐CNN group had the best survival outcomes. The differences in survival between the four groups are presented in Figure 3. Compared with the non‐anemia and non‐CNN group, the anemia group, CNN group, anemia and CNN group experienced poorer OS, DMFS, LRRFS, and DFS. In addition, the anemia and CNN group had poorer OS, DMFS, LRRFS and DFS than the CNN group; and the anemia and CNN group had poorer DMFS and DFS than the anemia group. There was no significant difference in any end‐point between the anemia group and CNN group.

Multivariate analysis demonstrated that the combined classification was an independent prognostic factor for OS (*P *=* *0.034), DMFS (*P *=* *0.001), LRRFS (*P *<* *0.001), and DFS (*P *=* *0.001). WHO pathology, T category, N category, and age were significant prognostic factor for OS and DFS; N caterogy was an independent prognostic factor for OS; and WHO pathogory was an independent prognostic factor for LRRFS. In addition, the anemia and CNN group had much higher HRs for OS, DMFS, LRRFS, and DFS than the other three groups, whereas the non‐anemia and non‐CNN group had the lowest HRs for all endpoints (Table 4).

**Table 4:** Combined prognostic value of anemia and cervical node necrosis (CNN) for OS, DFS, DMFS and LRRFS in the 1302 patients with NPC

Endpoint	Variable		HR (95% CI)	*P*‐value^a^
OS	WHO pathology		0.34 (0.20–0.58)	<0.001
	CNN and anemia			0.034
		Non‐anemia and non‐CNN	Reference	
		Anemia	2.17 (1.03–4.57)	0.041
		CNN	1.48 (1.00–2.17)	0.048
		Anemia and CNN	2.42 (1.02–5.75)	0.046
	T category		3.29 (1.44–7.53)	0.003
	N category		1.55 (1.05–2.27)	0.022
	Age		2.01 (1.38–2.91)	<0.001
	Gender		NS	
	Chemotherapy		NS	
DFS	WHO pathology		0.49 (0.32–0.78)	0.003
	CNN combined with anemia			<0.001
		Non‐anemia and non‐CNN	Reference	
		Anemia	1.64 (0.88–3.06)	0.120
		CNN	1.64 (1.24–2.18)	0.001
		Anemia and CNN	4.09 (2.230–7.29)	0.000
	T category		1.98 (1.23–3.19)	0.005
	N category		1.34 (1.06–1.79)	0.046
	Age		1.47 (1.11–1.92)	0.004
	Gender		NS	
	Chemotherapy		NS	
DMFS	WHO pathology		NS	
	CNN combined with anemia			0.001
		Non‐anemia and non‐CNN	Reference	
		Anemia	1.29 (0.54–3.21)	0.591
		CNN	1.43 (0.99–2.08)	0.060
	T category		NS	
	N category		1.97 (1.37–2.82)	<0.001
		Anemia and CNN	4.29 (2.14–8.58)	<0.001
	Age		NS	
	Gender		NS	
	Chemotherapy		NS	
LRRFS	WHO pathology		0.34 (0.19–0.61)	<0.001
	CNN combined with anemia			<0.001
		Non‐anemia and non‐CNN	Reference	
		Anemia	2.19 (0.93–5.12)	0.071
		CNN	2.08 (1.39–3.13)	<0.001
		Anemia and CNN	5.2878 (2.37–11.74)	<0.001
	T category			
	N category			
	Age		NS	
	Gender		NS	
	Chemotherapy		NS	

NPC, nasopharyngeal carcinoma; HR, hazard ratio; CI, confidence interval; OS, overall survival; LRRFS, locoregional relapse‐free survival; DMFS, distant metastasis‐free survival; DFS, disease‐free survival; NS, not significant.

a The following parameters were included in the Cox proportional hazards model multivariate analysis: WHO pathology (Type I‐II vs. Type III), gender (male vs. female), age (>45 vs. ≤45 years), chemotherapy (yes vs. no), T category (T1–2 vs. T3–4), and N category (N1 vs. N2–3).

## Discussion

Radioresistance and chemoresistance are the major factors associated with poor locoregional control and distant failure in malignant tumors 8. Tumor hypoxia plays a major role in radioresistance and chemoresistance 9, 10. Recent evidence indicates both anemia and CNN are associated with tumor hypoxia, which negatively impact treatment efficacy 11, 12, 13, 14, 15, 16, 17. Therefore, a large population‐based study was performed to determine the combined prognostic value of anemia and CNN in current study. Based on this work, we propose a new prognostic model that enables accurate stratification of individual patients with NPC receiving IMRT ± chemotherapy into four risk groups.

This study revealed pretreatment anemia was a prognostic factor for poorer DMFS, and pretreatment CNN was a prognostic factor for poorer OS, DFS, DMFS, and LRRFS. Therefore, we sought to establish a prognostic model. The non‐anemia and non‐CNN group had better survival outcomes than the anemia group, CNN group, anemia and CNN group, and the anemia and CNN group experienced the poorest survival outcomes. The combined classification was an independent prognostic factor for OS, LRRFS, DMFS, and DFS. The anemia and CNN group had much higher HRs for all endpoints than the other three groups, indicating combined assessment of CNN and anemia improves risk stratification and prognostication and suggesting more intensive treatment modality may be more suitable for patients with pretreatment anemia and CNN.

The prognostic significance of low pretreatment anemia has previously been investigated in various cancers; low Hb is associated with poorer outcomes in many types of human cancer, particularly in HN cancers and gynecological tumors 26, 27, 28. A positive relationship between survival outcomes and Hb levels has been demonstrated in patients with NPC receiving RT ± chemotherapy: low pretreatment Hb was related to increased risk of death or metastasis 11, 12, 13, 14, 15.

Moreover, CNN has also been demonstrated to have prognostic value in HN cancer after RT and/or chemotherapy 29, 30, 31: the presence of hypodense lymph nodes, which indicate CNN 17, was associated with regional treatment failure. Lan et al. 14 were the first to report CNN was a negative prognostic factor for OS, DMFS, RRFS, and DFS in patients with NPC treated using IMRT, three‐dimensional radiotherapy (3D‐RT) or 2D‐RT. Moreover, in a recent study by Zhang et al., CNN was demonstrated to be an independent, adverse prognostic factor for OS, DFS, LRRFS, and DMFS in patients with NPC treated with IMRT 17.

The major purpose of this research was to combine the prognostic value of anemia and CNN to improve risk stratification and prognostication in NPC. We found the combination of anemia and CNN improved prognostic ability. However, the precise mechanisms underlying the cumulative adverse effect of anemia and CNN remain unclear. As both anemia and CNN are associated with hypoxia, we speculate the presence of both may indicate more severe tumor hypoxia compared to one factor alone. As there is a potential relationship between hypoxia and radioresistance and chemoresistance and the anemia and CNN group had the poorest prognosis compared to the other three groups, we suggest more aggressive IMRT ± chemotherapy protocols should be designed for high‐risk patients with anemia and CNN.

The principal limitation of this study was the fact treatment heterogeneity was inevitable as this was a retrospective analysis of patients with NPC from a single institution. Thus, a large, multicenter prospective study is required to further confirm the findings of current research.

## Conclusion

Classification combining anemia and CNN is an independent prognostic factor for OS, DFS, DMFS, and LRRFS in patients with NPC treated with IMRT ± chemotherapy. Combined assessment of anemia and CNN improves risk stratification and prognostication: patients in the non‐anemia and non‐CNN group had the best survival outcomes; and the anemia and CNN group experienced the poorest survival outcomes. This data may aid the design of individualized treatment plans to improve the treatment outcomes of patients with NPC receiving IMRT ± chemotherapy.

## Conflict of Interest

The authors declare that they have no competing interests.
